# Co-Occurring Alcohol Use Disorder and Post-Traumatic Stress Disorder

**DOI:** 10.35946/arcr.v39.2.01

**Published:** 2018

**Authors:** Robert M. Anthenelli, Kathleen T. Brady, Lindsey Grandison, Deidra Roach

**Affiliations:** Robert M. Anthenelli, M.D., is a professor and the executive vice chair for the Department of Psychiatry, University of California San Diego, La Jolla, California. Kathleen T. Brady, M.D., Ph.D., is a distinguished university professor, the vice president for research, and the director of the South Carolina Clinical and Translational Research Institute, Medical University of South Carolina, Charleston, South Carolina. Lindsey Grandison, Ph.D., National Institute on Alcohol Abuse and Alcoholism, Rockville, Maryland. Deidra Roach, M.D., is a medical project officer in the Division of Treatment and Recovery Research, National Institute on Alcohol Abuse and Alcoholism, Rockville, Maryland

**Figure:**
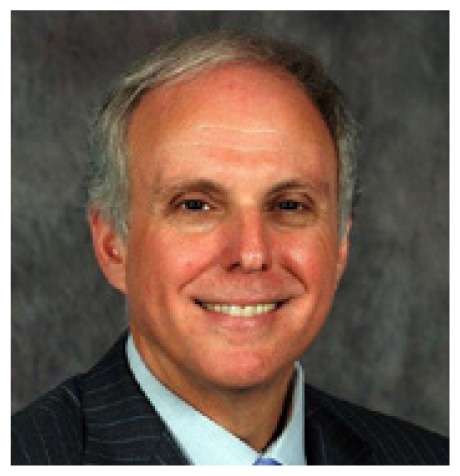
Robert M. Anthenelli

**Figure:**
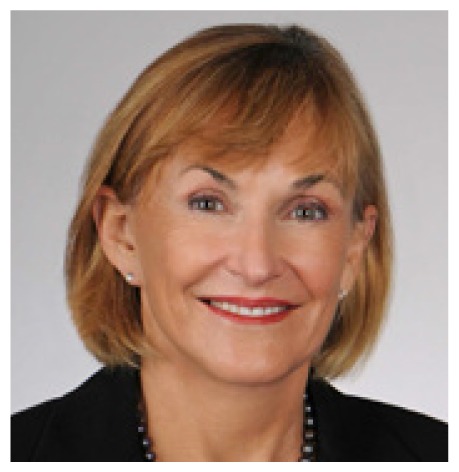
Kathleen T. Brady

**Figure:**
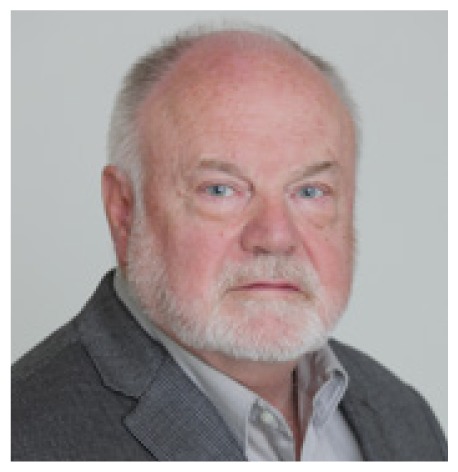
Lindsey Grandison

**Figure:**
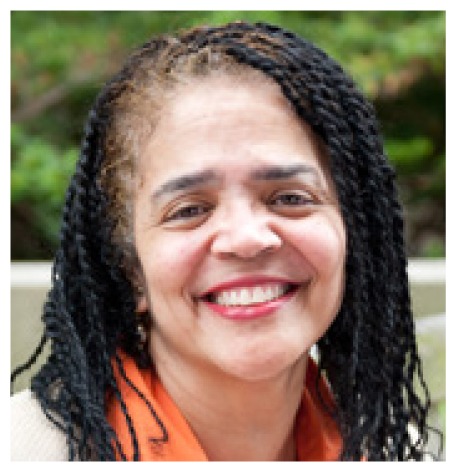
Deidra Roach

Alcohol use disorder (AUD) is a chronic, relapsing brain disease characterized by a reduced ability to stop or control alcohol use despite negative social, work, or health consequences. Often, it co-occurs and interacts with post-traumatic stress disorder (PTSD), which may develop after experiencing or witnessing a life-threatening event, such as combat, a natural disaster, a car accident, or sexual assault, and can result in shock, confusion, anger, and anxiety.

Co-occurring AUD and PTSD is a public health concern, especially among active military service members and veterans, as well as victims of violence and sexual assault. Approximately one in three people who have experienced PTSD have also experienced AUD at some point in their lives.^1^,^2^ In addition, 30% to 60% of patients seeking treatment for AUD also meet diagnostic criteria for PTSD.^3^,^4^ The co-occurrence of AUD and PTSD worsens adverse health outcomes and complicates treatment for both conditions.

This issue of *Alcohol Research: Current Reviews* examines the current literature on the prevalence, diagnoses, causes, and risk factors of AUD and PTSD, their co-occurrence, and treatment for individuals facing both conditions.

Smith and Cottler, in **The Epidemiology of Post-Traumatic Stress Disorder and Alcohol Use Disorder**, describe the changes in the *Diagnostic and Statistical Manual of Mental Disorders* (DSM) definitions of AUD and PTSD. They review key surveys that have measured these disorders, the possible relationships between the two disorders, the risk factors, and which populations are at risk.

In **Functional and Psychiatric Correlates of Comorbid Post-Traumatic Stress Disorder and Alcohol Use Disorder**, Straus and colleagues present the DSM-5 definitions for PTSD and AUD and discuss models for functional relationships between the disorders. They also examine risk factors and their associations with co-occurring disorders.

Suh and Ressler, in **Common Biological Mechanisms of Alcohol Use Disorder and Post-Traumatic Stress Disorder**, review animal models for and clinical studies of AUD and PTSD. They discuss the relevant neurobiological circuits and examine the role of stress in these disorders.

Lee and colleagues investigate childhood stress as a predictor for PTSD and AUD in **Early Life Stress as a Predictor of Co-Occurring Alcohol Use Disorder and Post-Traumatic Stress Disorder**. They review both human and preclinical models of these disorders and examine potential biologic, genetic, and epigenetic mechanisms.

In **Co-Occurring Post-Traumatic Stress Disorder and Alcohol Use Disorder in U.S. Military and Veteran Populations**, Dworkin and colleagues report on the frequency of co-occurring PTSD and AUD in military personnel and veterans, and they examine population-specific factors contributing to the development of PTSD and AUD. They also describe evidence-based psychological and pharmacological treatments for these populations and suggest future directions for research on treatment effectiveness.

Weil and colleagues provide an overview of the bidirectional relationships between traumatic brain injury and AUD in **Alcohol Use Disorder and Traumatic Brain Injury**. The potential neuropsychological and neurobiological mechanisms underlying those relationships are discussed.

In **Behavioral Treatments for Alcohol Use Disorder and Post-Traumatic Stress Disorder**, Flanagan and colleagues describe evidence-supported behavioral interventions for treating AUD, PTSD, and co-occurring AUD and PTSD. They also examine the debate regarding sequential versus integrated treatment models.

In **Pharmacotherapy for Co-Occurring Alcohol Use Disorder and Post-Traumatic Stress Disorder: Targeting the Opioidergic, Noradrenergic, Serotonergic, and GABAergic/Glutamatergic Systems**, Verplaetse and colleagues report on pharmacotherapies for co-occurring AUD and PTSD. They discuss current clinical trials for medications and highlight future directions for neurobiological targets that have potential for treating individuals with this dual diagnosis.
